# Menin inhibition in pediatric UBTF-TD myelodysplastic syndrome: molecular rationale and clinical observation

**DOI:** 10.1186/s40348-026-00264-x

**Published:** 2026-10-06

**Authors:** Franziska Rummel, Tabea Blessing, Triantafyllia Brozou, Sujal Ghosh, Roland Meisel, Maximilian Seidl, Arndt Borkhardt

**Affiliations:** 1 Department of Pediatric Oncology, Hematology and Clinical Immunology, Medical Faculty, Heinrich Heine University Düsseldorf, Düsseldorf, Germany; 2 Institute of Pathology, Medical Faculty, Heinrich Heine University Düsseldorf, Düsseldorf, Germany; 3 Department of Pediatric Oncology, Klinikum Dortmund, Dortmund, Germany

**Keywords:** Pediatric MDS, UBTF-TD, Menin inhibition, Revumenib, Childhood cancer, HSCT

## Abstract

**Background:**

UBTF tandem duplications (UBTF-TD) define a recently recognized high-risk subtype of pediatric myelodysplastic syndrome (MDS) and acute myeloid leukemia (AML). UBTF-TD myeloid neoplasms are driven by a HOX/MEIS1 transcriptional program that depends on the interaction between menin and KMT2A. Preclinical studies have shown sensitivity to menin inhibition, but clinical experience in children with MDS remains scarce.

**Results:**

We report a ten-year-old girl with MDS with excess blasts harboring UBTF-TD together with NRAS and WT1 mutations. During the first cycle of azacitidine and venetoclax, blood counts did not improve, and the patient remained dependent on platelet and red blood cell transfusions. Revumenib was added to the ongoing treatment. Within two weeks, platelet counts and hemoglobin increased markedly, the patient became transfusion independent, and bone marrow blasts cleared. UBTF-TD also became undetectable by molecular testing. The patient subsequently underwent allogeneic hematopoietic stem cell transplantation and remains in complete remission with full donor chimerism one year after transplantation.

**Conclusions:**

The rapid hematologic recovery and clearance of both bone marrow blasts and detectable UBTF-TD after initiation of revumenib provide clinical evidence that the menin dependency of UBTF-TD myeloid neoplasms can be therapeutically exploited. The timing and depth of the clinical and molecular response were remarkable.

## Background

Pediatric myelodysplastic syndromes (MDS), particularly MDS with excess blasts (MDS-EB), are increasingly stratified by their underlying molecular alterations rather than morphology alone. The upstream binding transcription factor UBTF has recently gained interest in hematological malignancies. *UBTF* encodes the upstream binding transcription factor, a cytoarchitectural protein essential for ribosomal RNA (rRNA) transcription, processing, and regulating the chromatin structure of ribosomal DNA [1]. Recurrent exon 13 tandem duplications (TD) in *UBTF* lead to a gain-of-function alteration resulting in mislocalization to genomic loci, including the *HOXA/HOXB* gene clusters and *MEIS1* [2]. UBTF-TD occupies these key genomic loci together with KMT2A and menin, linking it mechanistically to the menin-KMT2A transcriptional axis and the HOX/MEIS1-driven leukemic program [2]. Clinically, UBTF-TD is associated with aggressive clinical behavior in myeloid neoplasms and a substantial risk of progression to acute myeloid leukemia (AML) [3, 4]. As a high-risk subtype of pediatric AML [5], it exhibits poor response to conventional chemotherapy and MRD positivity [6]. The prognostic impact is severe: in a Japanese AML cohort, pediatric patients with UBTF-TD had a three-year event-free survival (EFS) of only 20% compared to 55% in *UBTF*-wildtype patients, and a three-year overall survival (OS) of 40% versus 74% [7]. MDS with UBTF-TD is prognostically equivalent to AML with UBTF-TD and appears to be a biologic continuum of the same molecularly defined myeloid neoplasm, rather than separate disease entities [4]. In a German cohort, Erlacher et al. identified UBTF-TD in approximately a third of all pediatric patients with advanced primary MDS and absence of germline predisposition [8].

Preclinical data demonstrated that primary cells from UBTF-TD leukemias are sensitive to menin inhibition through disruption of HOX/MEIS-dependent transcriptional programs, resulting in myeloid differentiation [2]. Revumenib (SNDX-5613), an oral menin inhibitor, has shown clinical efficacy in KMT2A-rearranged leukemias and received FDA approval in November 2024 for relapsed or refractory acute leukemia with KMT2A translocation in patients aged ≥ 1 year [9].

## Results

A previously healthy ten-year-old girl of German origin presented in our hematology department in May 2025 with progressive fatigue, pallor and spontaneous ecchymoses and hematomas. Laboratory evaluation revealed severe anemia (hemoglobin 6.8 g/dL), profound thrombocytopenia (11,000/µL) and mild neutropenia (1,100/µL). She received frequent platelet and erythrocyte transfusions (transfusions were administered at hemoglobin levels < 7 g/dL and platelet counts < 20,000/µL).

**Fig. 1:**
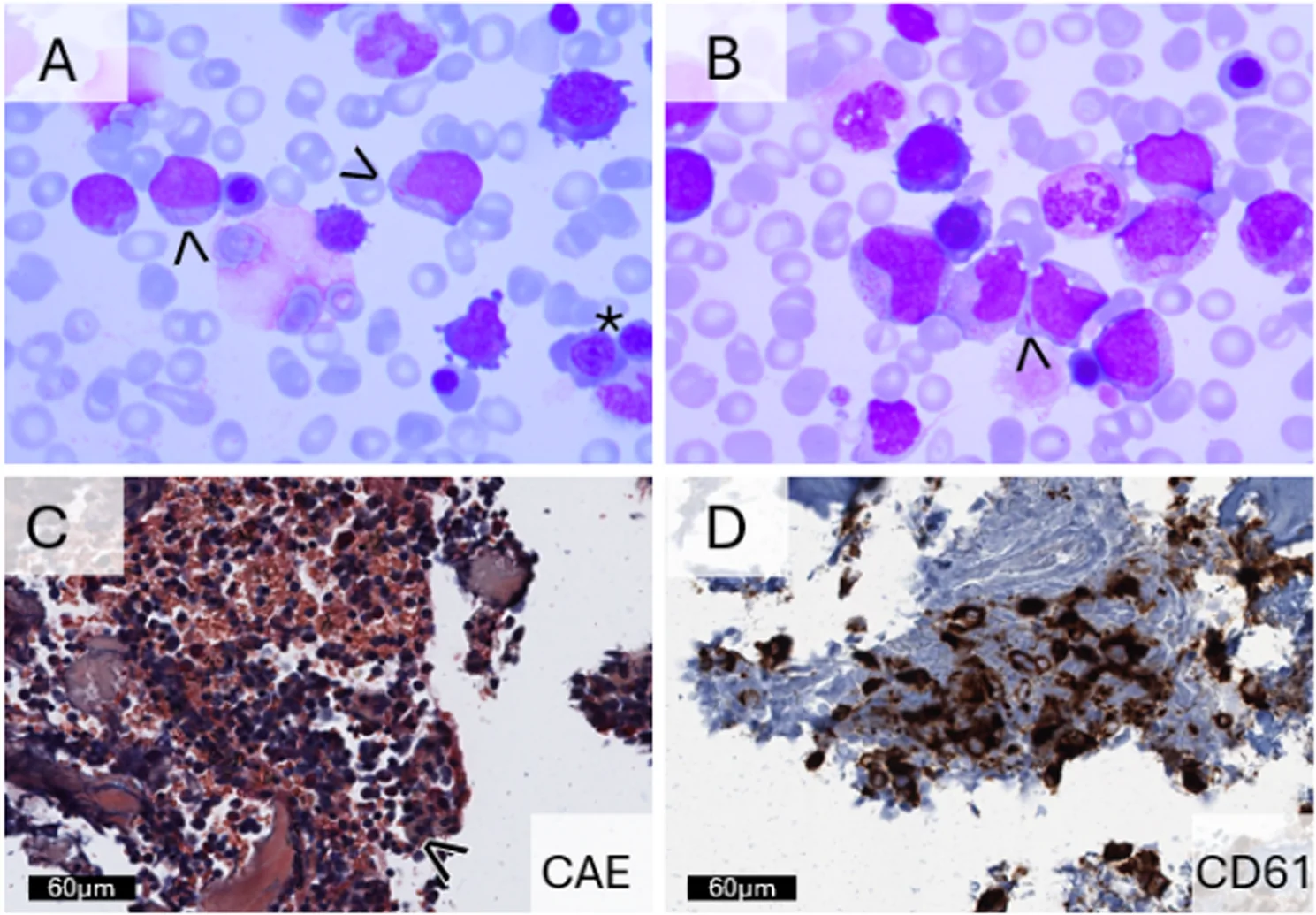
Bone marrow morphology and histology showing dysplasia and aberrant megakaryopoiesis: Bone marrow smear cytology: (**A**) Auer rods (marked by arrowheads) and irregular nuclear shape of erythropoietic cells (marked by asterisk). (**B**) Persistent dysplasia in the bone marrow smear 12 days after (**A**). (**C**) Histology (chloroacetate esterase staining (CAE)) displaying disturbed distribution with intermixed erythropoiesis, granulopoiesis and small, dysplastic megakaryocytes (marked by arrowhead). (**D**) CD61 immunohistochemistry displaying grouped, dysplastic megakaryocytes. **A**, **B**) scanned with 1000x original magnification with a Leica DM2500 microscope. **C**, **D**) scanned with 40x original magnification with a Leica Aperio slide scanner (Leica, Nussloch, Germany), scale bars are shown

Diagnostic work-up, including bone marrow biopsy and aspiration showed a markedly hypercellular marrow with severe multilineage dysplasia affecting the erythroid, granulocytic and megakaryocytic lineages. Myeloid blasts accounted for 18% of nucleated cells, and occasional Auer rods were present, consistent with myelodysplastic syndrome with excess blasts (MDS-EB) (Fig. 1).

Molecular analysis performed at the German AML reference laboratory identified a *UBTF* tandem duplication (UBTF-TD ratio (mutant/wildtype): 0.73), alongside with *WT1* and *NRAS* mutations. This molecular profile indicates a high-risk MDS with limited likelihood of longtime durable control. Allogeneic hematopoietic stem cell transplantation was therefore considered as the best curative treatment. Given the substantial number of blasts, adequate disease control before transplantation seemed highly desirable. Thus, our patient received a treatment cycle according to the European Working Group of MDS in Childhood (EWOG MDS) study recommendations with azacitidine and venetoclax. Azacitidine was administered intravenously at 100 mg/m² body surface area once daily for 5 consecutive days in combination with oral venetoclax, which was started at 50 mg/m^2^ body surface area once daily and escalated stepwise to a dose of 170 mg/m² body surface area once daily for 28 days. The leveraged dose of venetoclax was adapted due to simultaneous antifungal prophylaxis with the strong CYP3A4 inhibitor voriconazole and was subsequently modified following the switch to the moderate CYP3A4 inhibitor isavuconazole. Triazole antifungals have been shown to increase the blood concentration of venetoclax [10].

Although venetoclax/azacitidine achieved complete remission in a small case series of three pediatric UBTF-TD MDS-EB patients [11], our patient demonstrated no visible response to this regimen, with persistent transfusion dependency and no improvement in platelet count or hemoglobin. Bone marrow biopsy after completion of the first azacitidine/venetoclax cycle still showed dysplastic maturation abnormalities of megakaryopoiesis, granulopoietic hypoplasia and erythropoietic hyperplasia, though blasts were no longer detectable. We aimed to achieve better disease control prior to HSCT to potentially improve our patient’s prognosis.

**Fig. 2:**
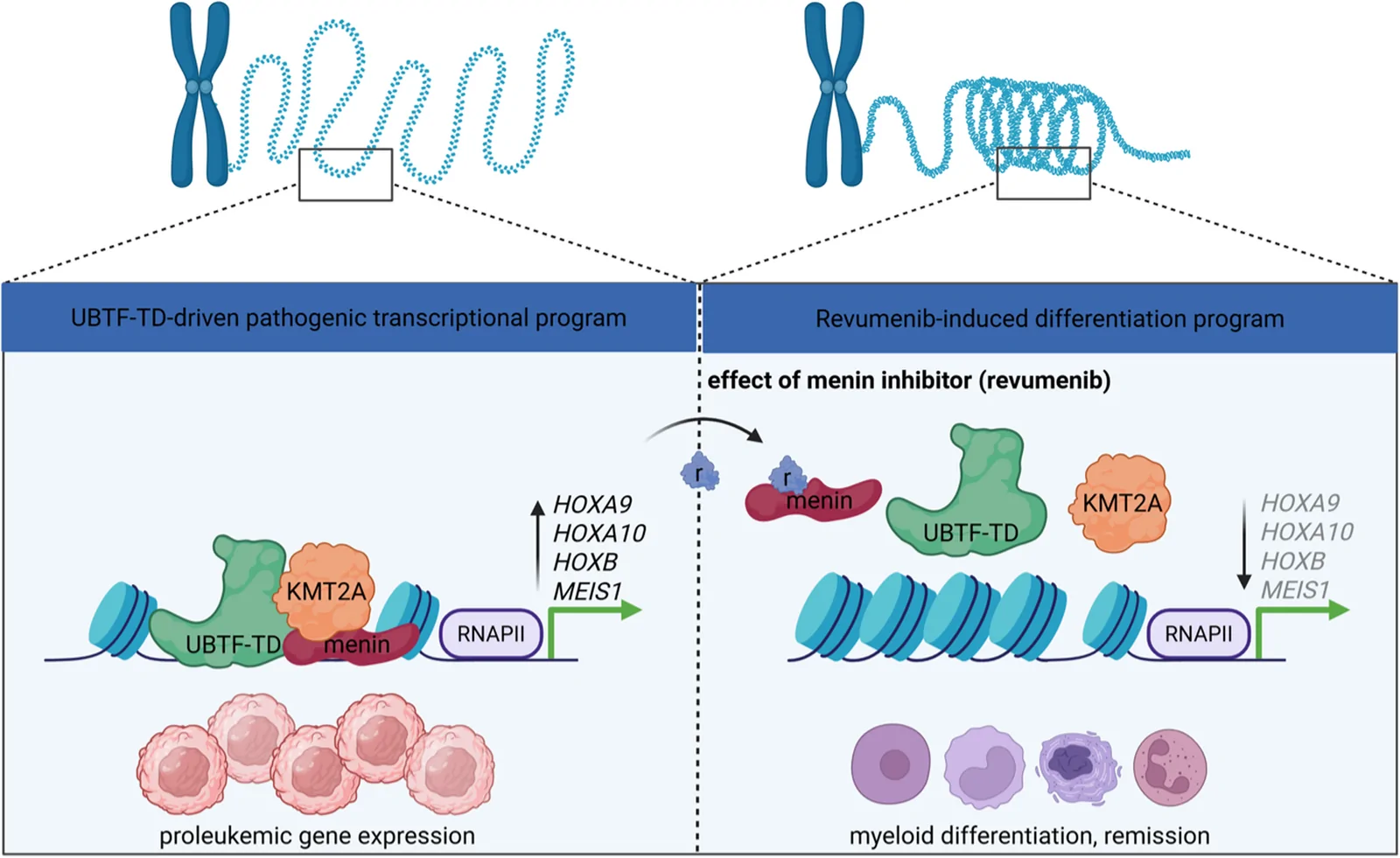
Schematic mechanism of UBTF-TD-driven leukemogenesis and revumenib-mediated inhibition inducing myeloid differentiation and remission. UBTF-TD promotes aberrant recruitment of the UBTF/menin/KMT2A complex to *HOXA/HOXB* and *MEIS1* loci, sustaining a proleukemic transcriptional program, which leads to proleukemic gene expression. Inhibition of the menin-KMT2A interaction by revumenib (SNDX-5613) disrupts this complex, suppresses HOX/MEIS1-associated transcription, and induces myeloid differentiation and remission. Created in https://BioRender.com

The mechanistic basis for menin inhibitor sensitivity in UBTF-TD has been demonstrated by Barajas et al. They showed that UBTF-TD and KMT2A/menin complex co-occupy genomic targets and the complex can efficiently be disrupted by menin inhibition [2] (Fig. 2).

We initiated menin inhibitor therapy with oral revumenib starting at 50 mg twice daily and stepwise increasing to a dose of 140 mg twice daily over 8 days. The treatment was well tolerated, with no differentiation syndrome, QTc prolongation or other significant adverse events during the four weeks of treatment.

**Fig. 3:**
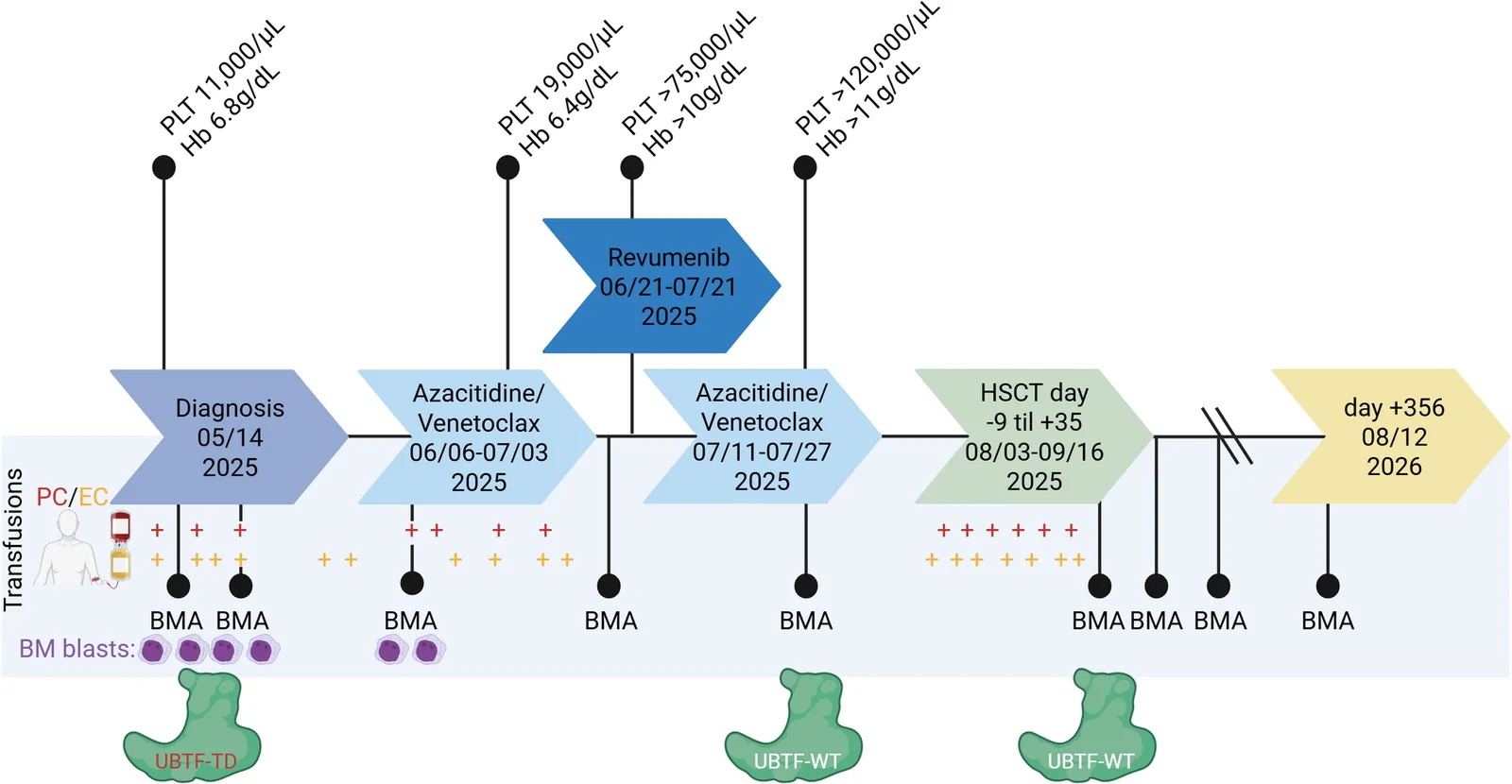
Visualization of clinical timeline of diagnosis, treatment, transfusion support, response, transplantation and follow-up. At initial presentation, the patient was diagnosed with MDS-EB with UBTF-TD. Bone marrow aspiration (BMA) confirmed excess blasts (May 15: 18% blasts, May 26: 19% blasts, June 5: 13% blasts). The patient required ongoing transfusions with platelet concentrates (PC) and erythrocyte concentrates (EC) (in the figure, each “+” indicates either one erythrocyte concentrate or one platelet concentrate). An initial treatment cycle with azacitidine/venetoclax was administered. During this period the patient showed neither relevant hematologic improvement nor reduction in transfusion frequency. BM biopsy after the first azacitidine/venetoclax cycle still showed dysplastic features, though blasts were no longer detectable. Two weeks after initiation of revumenib, the platelet count and hemoglobin level elevated and no more transfusions were required. BMA after end of the revumenib treatment showed molecular clearance of UBTF-TD. A second treatment cycle with azacitidine/venetoclax was administered. The patient subsequently underwent allogeneic HSCT and achieved sustained hematologic recovery. Created in https://BioRender.com

Revumenib treatment resulted in rapid hematologic recovery and complete morphologic remission within two weeks after initiation of treatment. Our patient became transfusion independent for red blood cell concentrates (EC) and platelet concentrates (TC). The last erythrocyte transfusion was administered 9 days after initiation of revumenib, and the last platelet transfusion 2 days after initiation. Two weeks after starting revumenib, the patient’s platelet count increased to > 75,000/µL and hemoglobin levels exceeded  10 g/dL. After one month of revumenib therapy, platelet counts exceeded  120,000/µL and hemoglobin levels increased to > 11 g/dL (Fig. 3). Bone marrow aspiration showed no morphologic evidence of blasts nor features of dysplasia, but reduced cellularity without marrow particles and megakaryocytes. The UBTF tandem duplication became undetectable by molecular means (Fig. 3). Although hematologic remission had been achieved, a second cycle of azacitidine/venetoclax was administered as recommended by the national experts of the EWOG-MDS committee.

Afterward, the patient underwent allogeneic peripheral blood stem cell transplantation from an HLA-matched (10/12) unrelated donor, with a CD34^+^ cell dose of 8.4 × 10^6^/kg body weight. The donor exhibited an antigen mismatch at the HLA-DR locus and a non-permissive mismatch in the graft-versus-host direction at the HLA-DP locus. The conditioning regimen consisted of busulfan [target AUC 75 mg h/L] on days -8 to -5, cyclophosphamide [2 × 60 mg/kg] on days  -3 to -2, and melphalan [1 × 140 mg/m²] on day − 1. GvHD prophylaxis included ATG Grafalon^®^ (Neovii Pharmaceuticals, Switzerland) [1, 19, 20, 20 mg/kg] on days  -5 to -2, cyclosporine A [2 × 1.5 mg/kg/day starting dose, target range: 120–150 ng/mL] from day − 1 until day + 60, and methotrexate [3 × 10 mg/m²] on days + 1, +3, and + 6. The HSCT was uneventful and the patient was discharged in excellent health status at day + 35. Post-transplant monitoring demonstrated complete donor chimerism (> 99% donor cells) in both bone marrow and peripheral blood. The bone marrow trephine biopsies at days + 30, +180 and + 365 showed normal findings and the patient remained molecularly negative for the *UBTF* tandem duplication. At the time of writing the patient remains in sustained remission with ongoing complete donor chimerism.

## Discussion

This case adds clinical evidence to the emerging concept that myeloid neoplasms with UBTF-TD may be therapeutically vulnerable to menin inhibition. UBTF-TD represents one of the most prognostically unfavorable molecular subtypes in pediatric AML, characterized by poor response to conventional chemotherapy, high rates of MRD positivity, and frequent relapse, which underscores the urgent need for molecularly targeted approaches [2, 5–7].

Primary cells from UBTF-TD leukemias showed sensitivity to revumenib, with reduced tumor growth, myeloid differentiation and abrogation of the leukemic expression signature [2]. This provides a plausible explanation for the rapid response observed in our patient, which is consistent with a transcriptional reprogramming effect.

Merli et al. recently reported that venetoclax/azacitidine achieved complete remission in three pediatric UBTF-TD MDS-EB patients [11]. The lack of response to venetoclax/azacitidine in our patient, followed by rapid response to revumenib, suggests that individual patients exhibit differential sensitivity to therapeutic approaches, potentially related to clonal heterogeneity or a specific co-mutational landscape. Notably, the presence of *WT1* co-mutation may have contributed to venetoclax resistance, as this mutation is associated with aggressive disease and unfavorable treatment response [3, 12].

This report has limitations. It describes only a single patient, limiting the generalizability of the findings, and prior azacitidine/venetoclax exposure may have contributed to later disease control. Nonetheless, the combination of morphologic remission, rapid transfusion independency and complete molecular clearance of the UBTF-TD is remarkable.

## Conclusions

This case provides preliminary clinical evidence that UBTF-TD-associated pediatric MDS - despite its inherently poor prognosis - may exhibit a therapeutically exploitable dependency on menin-mediated transcriptional regulation. These findings support further clinical trials of menin inhibition in UBTF-TD-associated pediatric myeloid neoplasms.

## Data Availability

No datasets were generated or analysed during the current study.

## Abbreviations

AML: Acute myeloid leukemia
BMA: Bone marrow aspiration
CD: Cluster of Differentiation
EC: Erythrocyte concentrate
EFS: Event-free survival
EWOG: European Working Group of MDS in Childhood
FDA: Food and Drug Administration
Hb: Hemoglobin
HOX: Homeobox
HSCT: Hematopoietic stem cell transplantation
KMT2A: Lysine methyltransferase 2 A (formerly MLL)
MDS: Myelodysplastic syndrome
MDS-EB: Myelodysplastic syndrome with excess blasts
MEIS1: Meis homeobox 1
MRD: Measurable residual disease
NRAS: Neuroblastoma RAS viral oncogene homolog
OS: Overall survival
PC: Platelet concentrate
PLT: Platelets
rRNA: Ribosomal RNA
TD: Tandem duplication
UBTF: Upstream binding transcription factor
UBTF-TD: Upstream binding transcription factor tandem duplication
UBTF-WT: Upstream binding transcription factor wildtype
WT1: Wilms tumor 1
